# Association of Nontraditional Lipid Indices With Poor Glycemic Control in Patients With Type 2 Diabetes: A Cross‐Sectional Study

**DOI:** 10.1155/jdr/6993567

**Published:** 2026-09-08

**Authors:** Amirhossein Yadegar, Fatemeh Mohammadi, Shima Loni, Sepideh Yadegar, Ali Mohammadi Naeini, Amirhossein Tayebi, Mahsa Abbaszadeh, Soghra Rabizadeh, Alireza Esteghamati, Manouchehr Nakhjavani, Sahar Karimpour Reyhan

**Affiliations:** ^1^ Endocrinology and Metabolism Research Center (EMRC), Vali-Asr Hospital, Tehran University of Medical Sciences, Tehran, Iran, tums.ac.ir; ^2^ Cardiovascular Research Center, Alborz University of Medical Sciences, Karaj, Iran, abzums.ac.ir

**Keywords:** dyslipidemia, glycated hemoglobin, HDL cholesterol, LDL cholesterol, triglycerides, Type 2 diabetes

## Abstract

**Background:**

This study investigated the association between poor glycemic control, defined as HbA1c ≥ 7.5%, and multiple nontraditional lipid indices, including AC, AIP, LCI, TG/HDL‐C ratio, CRI‐I, and CRI‐II, and to evaluate their diagnostic performance for identifying poor glycemic control.

**Methods:**

In this cross‐sectional study, 6424 individuals diagnosed with Type 2 diabetes (T2D) who visited a diabetes clinic from 2014 to 2024 were included. The relationships between nontraditional lipid indices and poor glycemic control were evaluated using RCS models and multivariable logistic regression. Diagnostic performance was examined using ROC analysis.

**Results:**

Nontraditional lipid indices were significantly elevated in patients with poor glycemic control (*p* < 0.001). In RCS models, significant nonlinear associations were observed between all lipid indices and poor glycemic control, with progressively higher odds of HbA1c ≥ 7.5% as index values increased (*p* for nonlinearity < 0.05). When analyzed as continuous variables, all indices were positively associated with poor glycemic control, with AIP showing the strongest association (OR = 2.25 [1.84–2.76]). Individuals in higher quartiles of each lipid index had significantly greater odds of poor glycemic control compared with those in the first quartile. All indices showed modest discriminatory ability (AUCs ≥ 0.684) for poor glycemic control, with AIP demonstrating the highest AUC (0.687 [0.657–0.718]). However, no statistically significant differences in AUC were detected among the indices.

**Conclusions:**

The observed nonlinear associations between nontraditional lipid indices and poor glycemic control highlight the close interplay between atherogenic dyslipidemia and glycemic dysregulation. As these indices are derived from routinely measured lipid parameters, they can act as practical complementary tools for identifying individuals at higher risk of poor glycemic control, especially in settings where HbA1c measurement is limited or not cost‐effective. Further research is required to confirm these findings and determine causal relationships.

## 1. Introduction

As per projections from the International Diabetes Federation (IDF), it is anticipated that the worldwide prevalence of diabetes will approach almost 700 million by the year 2045 [1]. This rising burden poses significant public health challenges, as individuals with diabetes experience reduced life expectancy in comparison to the general population and are at heightened risk for chronic complications, particularly cardiovascular disease, which accounts for considerable morbidity and mortality [2–4]. Dyslipidemia is a frequent metabolic disorder among patients with Type 2 diabetes (T2D), and poor glycemic control is closely correlated with dyslipidemia marked by high triglycerides (TG) and low‐density lipoprotein cholesterol (LDL‐C) levels, along with decreased high‐density lipoprotein cholesterol (HDL‐C) [5–8].

Beyond the traditional lipid profile, multiple novel lipid indices, such as the atherogenic coefficient (AC), atherogenic index of plasma (AIP), lipoprotein combine index (LCI), TG/HDL‐C ratio, Castelli risk index I (CRI‐I), and Castelli risk index II (CRI‐II), have been introduced in recent years as potential markers for cardiometabolic risk stratification [9–12]. These indices are derived from routinely measured lipid parameters, are easy to calculate in medical practice, and may offer a more thorough assessment of atherogenic dyslipidemia than individual lipid measures alone [10].

Despite recent advances in diabetes management, poor glycemic control remains a major challenge among individuals with diabetes [13]. Hemoglobin A1c (HbA1c) is commonly utilized as an indicator of long‐term glycemic management and is a well‐established predictor of diabetes‐related complications, outperforming fasting plasma glucose (FPG) in this respect [14]. In addition to its role in glycemic monitoring, HbA1c has been linked to dyslipidemia and cardiovascular risk [15]. Higher HbA1c levels are connected with the degree of insulin resistance, progressive *β*‐cell dysfunction, and atherogenic lipid abnormalities [16, 17]. Despite its importance, in many regions worldwide, HbA1c testing remains inaccessible or expensive, limiting its routine clinical use, particularly in resource‐limited settings [14].

Earlier studies have reported associations between elevated HbA1c levels and higher values of AIP, AC, and the TG/HDL‐C ratio, reflecting a predominance of atherogenic dyslipidemia in patients with T2D [18–20]. Nevertheless, evidence remains limited regarding a comprehensive evaluation of the relationship between poor glycemic control—defined as HbA1c ≥ 7.5% [21]—and nontraditional lipid indices, as well as direct comparisons of their performance as indicators of atherogenic dyslipidemia. Therefore, this study sought to investigate the association between poor glycemic control (HbA1c ≥ 7.5%) and several novel lipid indices, including AC, AIP, LCI, TG/HDL‐C ratio, CRI‐I, and CRI‐II. Additionally, the present study assessed the diagnostic performance of these indices in identifying poor glycemic control, with particular relevance for settings in which HbA1c measurement is not attainable or cost‐effective.

## 2. Materials and Methods

### 2.1. Study Population

This cross‐sectional research included individuals with T2D who visited the diabetes clinic of a tertiary hospital affiliated with Tehran University of Medical Sciences from 2014 to 2024. Exclusion criteria included conditions that could potentially alter lipid profiles and confound the results, such as liver cirrhosis, cancer, thyroid disorders, smoking, pregnancy, or the use of antioxidant supplements or oral contraceptives. The study population was relatively homogeneous; most participants were of middle socioeconomic status, had middle to high school education, and had access to healthcare services and health insurance coverage. Medication adherence was generally consistent across the study population. The study adhered to the principles of the Declaration of Helsinki and received approval from the Research Ethics Committee at Tehran University of Medical Sciences (Approval number: IR.TUMS.IKHC.REC.1398.270). Informed consent in writing was obtained from every participant. No artificial intelligence tool was used in developing any portion of this manuscript.

### 2.2. Clinical and Laboratory Measurements

Demographic and clinical details, such as age, gender, diabetes duration, medical history, and medication use, were gathered from medical records and participant interviews. Weight was measured in light clothing employing a digital scale (Tefal PP1100). Height and waist circumference (WC) were assessed using a flexible measuring tape, with WC determined at the midpoint between the bottom edge of the rib cage and the top of the iliac crest. Body mass index (BMI) was calculated by dividing weight (kg) by the square of height (m^2^), and the waist‐to‐height ratio (WHtR) was calculated by dividing WC by height.

Systolic blood pressure (SBP) and diastolic blood pressure (DBP) were assessed by trained staff using a calibrated digital sphygmomanometer (Omron M7, Hoofddorp, The Netherlands). Measurements were obtained after a 5‐min rest, on the right arm positioned at heart level, and repeated after 10 min. The average of the two readings was recorded for SBP and DBP.

Blood samples from veins were obtained after a 12‐h fast. FPG and 2‐h postprandial glucose (2hPG), measured 2 h after a standard meal, were evaluated by the glucose oxidase technique (BT3000 Plus, Biotecnica, Italy). Serum levels of TG, total cholesterol (TC), LDL‐C, HDL‐C, and creatinine were analyzed using enzymatic methods (BT3000 Plus, Biotecnica, Italy). HbA1c values were measured using high‐performance liquid chromatography (HPLC) (DS5, Drew Scientific, United Kingdom).

Non‐HDL‐C was calculated as TC minus HDL‐C. Estimated glomerular filtration rate (eGFR) was determined using the CKD‐EPI equation from the Chronic Kidney Disease Epidemiology Collaboration [22]. Poor glycemic control was defined as HbA1c ≥ 7.5% [21]. Hypertension was characterized by SBP ≥ 140 mmHg, DBP ≥ 90 mmHg, or current use of antihypertensive medications [23].

The following formulas were used to calculate nontraditional lipid indices:

AC=TC−HDL−C/HDL−C

AIP=log TG/HDL−C

LCI=TC×TG×LDL−C/HDL−C

CRI−I=TC/HDL−C

CRI−II=LDL−C/HDL−C.

### 2.3. Statistical Analysis

The Kolmogorov–Smirnov test, along with visual inspections of P‐P plots and histograms, was used to assess the normality of the variables. Variables that follow a normal distribution were expressed as mean ± standard deviation (SD), whereas those that do not conform to a normal distribution were reported using median (Q1, Q3). Categorical variables were presented as frequencies and percentages. Comparisons between individuals with HbA1c < 7.5% and those with HbA1c ≥ 7.5% were performed using Student′s *t*‐test, Mann–Whitney *U* test, or chi‐square test, depending on what was suitable.

To investigate the association between nontraditional lipid indices and poor glycemic control (HbA1c ≥ 7.5%), restricted cubic spline (RCS) models and multivariable logistic regression were applied. RCS models were constructed with three knots positioned at the 10th, 50th, and 90th percentiles, using the median value of each lipid index as the reference point (odds ratio [OR] = 1). *P* values for nonlinearity were calculated to assess the presence of nonlinear associations. Multivariable logistic regression analyses were conducted to examine the association between HbA1c ≥ 7.5% and both continuous and categorical (quartile‐based) forms of each lipid index. Quartiles were compared with the first quartile as the reference group. Results were reported as ORs with 95% confidence intervals (CIs).

Receiver operating characteristic (ROC) curves were utilized to evaluate the diagnostic performance of each lipid index in identifying poor glycemic control. The area under the curve (AUC) and the associated 95% CIs were reported. Optimal cut‐off values were established using Youden′s index, and the associated sensitivity, specificity, and accuracy were determined. Comparisons of AUCs among lipid indices were performed using the DeLong test.

As a sensitivity analysis, all primary analyses were repeated using an HbA1c threshold of ≥ 7.0%, consistent with the glycemic target recommended by the ADA for most nonpregnant adults [24].

Covariates included in the models were selected individually for each lipid index based on variables that differed significantly between HbA1c groups and were not components of the corresponding index. All models were adjusted for diabetes duration, hypertension, SBP, DBP, lipid‐lowering agents, and diabetes medications. Additional specific covariates included WHtR and TG for AC; WHtR and LDL‐C for AIP and TG/HDL‐C; and WHtR for LCI. Statistical analyses were performed using Python (Version 3.12) with Pandas (Version 2.1.4), NumPy (Version 1.26), Matplotlib (Version 3.8.1), Scikit‐learn (Version 1.4.0), and SciPy (Version 1.13.1) libraries [25–29].

## 3. Results

### 3.1. Baseline Characteristics of the Study Population

This study enrolled 6424 individuals with T2D, with a mean age of 57.7 ± 10.6 years. The median diabetes duration was 8 years (Q1–Q3: 4–14), and 56.6% were women (*n* = 3633) (Table 1). Compared with patients with HbA1c < 7.5%, those with HbA1c ≥ 7.5% had a higher median diabetes duration (10 vs. 6 years, *p* < 0.001), higher mean WHtR (0.61 vs. 0.60, *p* = 0.045), SBP (131.9 vs. 129.0 mmHg, *p* < 0.001), DBP (79.2 vs. 78.5 mmHg, *p* = 0.001), FPG (191.4 vs. 135.4 mg/dL, *p* < 0.001), 2hPG (268.8 vs. 183.4 mg/dL, *p* < 0.001), TC (181.8 vs. 174.8 mg/dL, *p* < 0.001), LDL‐C (102.5 vs. 98.5 mg/dL, *p* < 0.001), non‐HDL‐C (137.2 vs. 129.7 mg/dL, *p* < 0.001), and median TG (157 vs. 141 mg/dL, *p* < 0.001) (Table 1). A greater proportion of patients with HbA1c ≥ 7.5% had hypertension (44.7% vs. 41.2%, *p* = 0.006) and were on multiple diabetes medications (41.8% vs. 27.4%, *p* < 0.001). All lipid indices, including AC, AIP, LCI, TG/HDL‐C ratio, CRI‐I, and CRI‐II, were significantly higher in patients with HbA1c ≥ 7.5% (all *p* < 0.001) (Table 1 and Table S1).

**Table 1:** Baseline characteristics of all participants and by HbA1c category.

Variable		Total (*n* = 6424)	HbA1c < 7.5% (*n* = 2967)	HbA1c ≥ 7.5% (*n* = 3457)	*p*
Age (years)		57.7 ± 10.6	57.9 ± 10.7	57.6 ± 10.4	0.328
Diabetes duration (years)		8 (4–14)	6 (3–12)	10 (5–15)	< 0.001
Women (*n*, %)		3633 (56.6%)	1696 (57.2%)	1937 (56.0%)	0.364
Hypertension (*n*, %)		2767 (43.1%)	1223 (41.2%)	1544 (44.7%)	0.006
BMI (kg/m^2^)		28.8 ± 4.7	28.8 ± 4.7	28.9 ± 4.7	0.767
WC (cm)		97.8 ± 10.2	97.6 ± 10.1	98.0 ± 10.3	0.061
WHtR		0.60 ± 0.06	0.60 ± 0.06	0.61 ± 0.07	0.045
SBP (mmHg)		130.5 ± 17.8	129.0 ± 17.0	131.9 ± 18.4	< 0.001
DBP (mmHg)		78.9 ± 9.4	78.5 ± 9.1	79.2 ± 9.6	0.001
FPG (mg/dL)		165.6 ± 61.1	135.4 ± 36.1	191.4 ± 66.2	< 0.001
2hPG (mg/dL)		229.3 ± 91.1	183.4 ± 62.6	268.8 ± 93.2	< 0.001
TG (mg/dL)		150 (108–209)	141 (103–195)	157 (112–223)	< 0.001
TC (mg/dL)		178.6 ± 44.6	174.8 ± 42.0	181.8 ± 46.5	< 0.001
LDL‐C (mg/dL)		100.6 ± 34.8	98.5 ± 33.1	102.5 ± 36.0	< 0.001
HDL‐C (mg/dL)		44.9 ± 11.1	45.1 ± 11.1	44.6 ± 11.1	0.065
Non‐HDL‐C (mg/dL)		133.7 ± 43.1	129.7 ± 40.7	137.2 ± 44.7	< 0.001
eGFR (mL/min/1.73m^2^)		77.8 ± 17.5	77.7 ± 17.2	77.9 ± 17.7	0.704
Lipid‐lowering agents (*n*, %)	Atorvastatin	5429 (84.5%)	2526 (85.1%)	2903 (84.0%)	0.390
	Rosuvastatin	801 (12.5%)	352 (11.9%)	449 (13.0%)	
	Fibrates	194 (3.0%)	89 (3.0%)	105 (3.0%)	
Diabetes medications (*n*, %)	Metformin monotherapy	3793 (59.0%)	2015 (67.9%)	1778 (51.4%)	< 0.001
	Sulfonylurea monotherapy	340 (5.3%)	130 (4.4%)	210 (6.1%)	
	Insulin monotherapy	33 (0.5%)	10 (0.3%)	23 (0.7%)	
	Multiple drug therapy	2258 (35.1%)	812 (27.4%)	1446 (41.8%)	
AC		3.15 ± 1.27	3.04 ± 1.23	3.24 ± 1.29	< 0.001
AIP		0.17 (0.01–0.35)	0.14 (−0.01–0.32)	0.19 (0.03–0.38)	< 0.001
LCI		14250 (9114–22509)	13340 (8694–20590)	15408 (9432–24430)	< 0.001
TG/HDL‐C ratio		3.40 (2.35–5.13)	3.18 (2.21–4.82)	3.58 (2.47–5.47)	< 0.001

*Note:* Continuous variables are presented as mean ± SD or median (Q1–Q3); categorical variables as *n* (%).

Abbreviations: 2hPG, 2‐h postprandial glucose; AC, atherogenic coefficient; AIP, atherogenic index of plasma; BMI, body mass index; DBP, diastolic blood pressure; eGFR, estimated glomerular filtration rate; FPG, fasting plasma glucose; HbA1c, hemoglobin A1c; HDL‐C, high‐density lipoprotein cholesterol; LCI, lipid combine index; LDL‐C, low‐density lipoprotein cholesterol; non‐HDL‐C, nonhigh‐density lipoprotein cholesterol; SBP, systolic blood pressure; TC, total cholesterol; TG, triglycerides; WC, waist circumference; WHtR, waist‐to‐height ratio.

### 3.2. RCS Model

**Figure 1:**
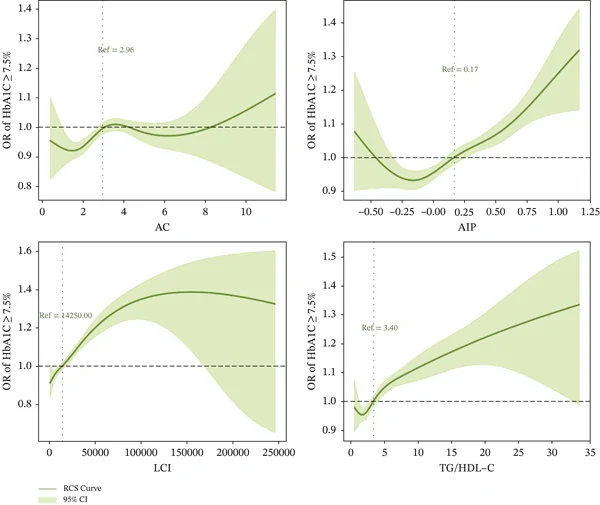
Adjusted RCS models with three knots at the 10^th^, 50^th^, and 90^th^ percentiles of each index. The models show significant nonlinear associations between all indices and HbA1c ≥ 7.5%, with higher index values associated with increased odds of HbA1c ≥ 7.5%. All models were adjusted for diabetes duration, WHtR, hypertension, SBP, DBP, lipid‐lowering agents, and diabetes medications. Additional covariates were as follows: AC (plus TG), AIP (plus LDL‐C), and TG/HDL‐C ratio (plus LDL‐C). RCS: restricted cubic spline; CI: confidence interval; OR: odds ratio; HbA1c: hemoglobin A1C; AC: atherogenic coefficient; AIP: atherogenic index of plasma; LCI: lipid combine index; TG: triglycerides; HDL‐C: high‐density lipoprotein cholesterol; SBP: systolic blood pressure; DBP: diastolic blood pressure; WHtR: waist‐to‐height ratio; LDL‐C: low‐density lipoprotein cholesterol.

RCS models were applied to evaluate possible nonlinear relationships between lipid indices and HbA1c ≥ 7.5%. The adjusted RCS models demonstrated significant nonlinear associations between all lipid indices and HbA1c ≥ 7.5%, showing higher odds of HbA1c ≥ 7.5% with increasing index levels (*p* for nonlinearity < 0.05) (Figure 1 and Figure S1) (Table 2 and Table S2).

**Table 2:** Odds ratios for selected knots in the restricted cubic spline models.

Lipid index	Selected knots			
	10^th^ percentile		90^th^ percentile	
	Value	Adjusted OR (95% CI)^a^	Value	Adjusted OR (95% CI)^a^
AC	1.75	0.93 (0.90–0.95)	4.81	0.99 (0.96–1.02)
AIP	−0.13	0.93 (0.91–0.96)	0.51	1.07 (1.04–1.10)
LCI	6175.8	0.96 (0.93–0.98)	34484.7	1.12 (1.09–1.14)
TG/HDL‐C ratio	1.68	0.95 (0.93–0.98)	7.43	1.08 (1.06–1.11)

Abbreviations: AC, atherogenic coefficient; AIP, atherogenic index of plasma; CI, confidence interval; DBP, diastolic blood pressure; HDL‐C, high‐density lipoprotein cholesterol; LCI, lipid combine index; LDL‐C, low‐density lipoprotein cholesterol; OR, odds ratio; SBP, systolic blood pressure; TG, triglycerides; WHtR, waist‐to‐height ratio.

^a^All models were adjusted for diabetes duration, WHtR, hypertension, SBP, DBP, lipid‐lowering agents, and diabetes medications. Additional covariates were as follows: AC (plus TG), AIP (plus LDL‐C), and TG/HDL‐C ratio (plus LDL‐C).

### 3.3. Association Between Continuous and Categorical Forms of Lipid Indices and HbA1c ≥ 7.5%

Multivariable logistic regression analyses were conducted to investigate the associations between both continuous and categorical (quartiles) forms of each lipid index and HbA1c ≥ 7.5%. After adjustment for covariates, all lipid indices in continuous form were significantly positively associated with HbA1c ≥ 7.5%, with AIP showing the highest odds ratio (OR = 2.25, 95% CI: 1.84–2.76) (Table 3 and Table S3). In the categorical analysis, higher quartiles were associated with higher odds of HbA1c ≥ 7.5%. The second, third, and fourth quartiles of AC, AIP, TG/HDL‐C ratio, and CRI‐I had significantly higher odds of HbA1c ≥ 7.5% compared with the first quartile. For LCI and CRI‐II, the third and fourth quartiles showed significant associations (Tables 3 and S3).

**Table 3:** Associations between lipid indices (continuous and categorical forms) and HbA1c ≥ 7.5%.

Lipid index		OR (95% CI) of HbA1c ≥ 7.5%	
		Unadjusted	Adjusted^a^
AC	Continuous (per one unit)	1.13 (1.09–1.18)	1.07 (1.02–1.12)
	Q1	Ref	
	Q2	1.26 (1.09–1.44)	1.25 (1.08–1.45)
	Q3	1.50 (1.30–1.72)	1.43 (1.24–1.66)
	Q4	1.51 (1.31–1.73)	1.26 (1.07–1.48)
AIP	Continuous (per one unit)	2.22 (1.83–2.69)	2.25 (1.84–2.76)
	Q1	Ref	
	Q2	1.21 (1.05–1.39)	1.22 (1.06–1.41)
	Q3	1.41 (1.23–1.62)	1.40 (1.21–1.61)
	Q4	1.75 (1.53–2.02)	1.78 (1.54–2.06)
LCI	Continuous (per 1000 unit)	1.02 (1.01–1.02)	1.02 (1.02–1.02)
	Q1	Ref	
	Q2	1.06 (0.92–1.22)	1.14 (0.99–1.32)
	Q3	1.16 (1.01–1.33)	1.25 (1.08–1.44)
	Q4	1.70 (1.48–1.96)	1.89 (1.63–2.19)
TG/HDL‐C ratio	Continuous (per one unit)	1.07 (1.05–1.09)	1.07 (1.05–1.09)
	Q1	Ref	
	Q2	1.23 (1.07–1.41)	1.24 (1.08–1.43)
	Q3	1.46 (1.27–1.67)	1.45 (1.26–1.68)
	Q4	1.75 (1.52–2.01)	1.77 (1.53–2.04)

Abbreviations: AC, atherogenic coefficient; AIP, atherogenic index of plasma; CI, confidence interval; DBP, diastolic blood pressure; HbA1c, hemoglobin A1c; HDL‐C, high‐density lipoprotein cholesterol; LCI, lipid combine index; LDL‐C, low‐density lipoprotein cholesterol; OR, odds ratio; SBP, systolic blood pressure; TG, triglycerides; WHtR, waist‐to‐height ratio.

^a^All models were adjusted for diabetes duration, WHtR, hypertension, SBP, DBP, lipid‐lowering agents, and diabetes medications. Additional covariates were as follows: AC (plus TG), AIP (plus LDL‐C), and TG/HDL‐C ratio (plus LDL‐C).

### 3.4. ROC Analysis

**Figure 2:**
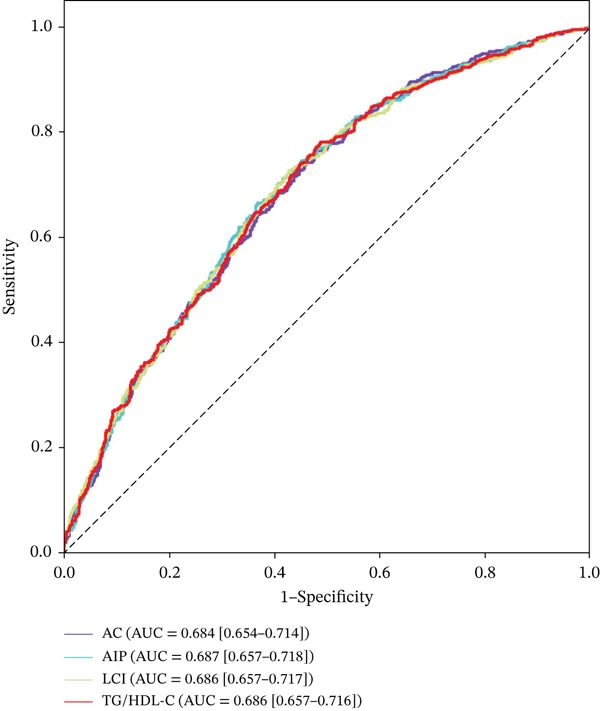
ROC curve analysis showing discriminative ability of lipid indices for HbA1c ≥ 7.5%. AIP had the highest AUC. All models were adjusted for diabetes duration, WHtR, hypertension, SBP, DBP, lipid‐lowering agents, and diabetes medications. Additional covariates were as follows: AC (plus TG), AIP (plus LDL‐C), and TG/HDL‐C ratio (plus LDL‐C). ROC: receiver operating characteristic; AUC: area under the curve; HbA1c: hemoglobin A1C; AC: atherogenic coefficient; AIP: atherogenic index of plasma; LCI: lipid combine index; TG: triglycerides; HDL‐C: high‐density lipoprotein cholesterol; SBP: systolic blood pressure; DBP: diastolic blood pressure; WHtR: waist‐to‐height ratio; LDL‐C: low‐density lipoprotein cholesterol.

ROC analysis was conducted to examine the discriminative ability of lipid indices for HbA1c ≥ 7.5%. After adjustment, all indices showed modest discrimination, with AUCs ≥ 0.684 (Figure 2 and Figure S2). AIP demonstrated the highest AUC (0.687). Among the indices, AIP had the highest specificity (63%), whereas the TG/HDL‐C ratio had the highest sensitivity (78%) (Table 4 and Table S4). The DeLong test revealed no significant differences between any pair of indices, indicating that no index was superior in identifying HbA1c ≥ 7.5%.

**Table 4:** Summary of ROC analysis for identifying HbA1c ≥ 7.5%.

Lipid indices	Cutoff	AUC (95% CI)^a^	Sensitivity	Specificity	Accuracy	*p*
AC	2.98	0.684 (0.654–0.714)	76%	52%	65%	< 0.001
AIP	0.22	0.687 (0.657–0.718)	67%	63%	65%	< 0.001
LCI	15344	0.686 (0.657–0.717)	73%	57%	66%	< 0.001
TG/HDL‐C ratio	3.60	0.686 (0.657–0.716)	78%	51%	66%	< 0.001

Abbreviation: AC, atherogenic coefficient; AIP, atherogenic index of plasma; AUC, area under the curve; CI, confidence interval; DBP, diastolic blood pressure; HbA1c, hemoglobin A1c; HDL‐C, high‐density lipoprotein cholesterol; LCI, lipid combine index; LDL‐C, low‐density lipoprotein cholesterol; ROC, receiver operating characteristic; SBP, systolic blood pressure; TG, triglycerides; WHtR, waist‐to‐height ratio.

^a^All models were adjusted for diabetes duration, WHtR, hypertension, SBP, DBP, lipid‐lowering agents, and diabetes medications. Additional covariates were as follows: AC (plus TG), AIP (plus LDL‐C), and TG/HDL‐C ratio (plus LDL‐C).

### 3.5. Sensitivity Analysis

Sensitivity analyses using HbA1c ≥ 7.0% showed findings that were consistent with the primary analyses (Tables S5–S7 and Figures S3–S6).

## 4. Discussion

This study evaluated the association between poor glycemic control, defined as HbA1c ≥ 7.5%, and several nontraditional lipid indices and assessed their ability to identify poor glycemic control. This study revealed significant nonlinear associations between all lipid indices and poor glycemic control. Overall, higher values of each index were associated with increased odds of HbA1c ≥ 7.5%. However, the strength of these associations varied across the range of index values, indicating nonlinear dose–response relationships. All lipid indices, when analyzed as continuous variables, were significantly associated with poor glycemic control, with AIP showing the strongest association. Additionally, individuals in higher quartiles of each lipid index had significantly greater odds of poor glycemic control compared with those in the first quartile. All indices showed modest discriminatory ability (AUCs ≥ 0.684) for poor glycemic control, with AIP demonstrating the highest AUC. However, no statistically significant differences were observed among the indices in terms of AUC.

Earlier studies have investigated the association between lipid indices and poor glycemic control or HbA1c levels. However, their findings have been inconsistent. In line with our results, a study conducted in Turkey reported higher AIP values among individuals with poor glycemic control, defined as HbA1c > 9%, and demonstrated positive correlations between HbA1c levels and AIP [30]. Similarly, higher TG/HDL‐C ratios have been observed among individuals with HbA1c ≥ 7%, and this index has been shown to correlate positively with HbA1c levels [20]. A study conducted in Indonesia reported significant positive correlations between HbA1c levels and CRI‐I, CRI‐II, and the TG/HDL‐C ratio. In that study, CRI‐I and CRI‐II were identified as risk factors for poor glycemic control (HbA1c > 7%), with CRI‐II showing the strongest association, whereas the TG/HDL‐C ratio was not significantly associated with poor glycemic control [31]. Consistently, another study found a significant positive correlation between HbA1c and CRI‐II, with individuals having HbA1c > 7% showing significantly higher CRI‐II values than those with HbA1c ≤ 7% [32]. In contrast, a study conducted in India reported no significant associations between several lipid indices, including AC, AIP, the TG/HDL‐C ratio, CRI‐I, and CRI‐II, and poor glycemic control, defined as HbA1c > 8% [33]. The discrepancies across studies may be primarily because of differences in the definitions of poor glycemic control, study populations, and sample sizes. In addition, regional variations in genetic background, dietary habits, socioeconomic status, and medication use may differently influence lipid metabolism and, consequently, lipid indices [6]. Overall, most previous studies have focused on individual lipid parameters or a single nontraditional lipid index, with limited efforts to comprehensively compare multiple lipid indices or evaluate their diagnostic performance for poor glycemic control. In this context, our research builds upon the current body of literature by providing a systematic comparison of several nontraditional lipid indices and assessing their discriminatory ability for identifying poor glycemic control. Nonetheless, further research, particularly in diverse populations and regions, is needed to confirm these findings and to elucidate the clinical utility of lipid indices in the assessment of poor glycemic control.

Dyslipidemia is highly prevalent among patients with T2D, even in the presence of adequate glycemic control, and is characterized by elevated TG and LDL‐C, along with decreased HDL‐C levels [6, 8]. Poor glycemic control further exacerbates lipid abnormalities in this population by disrupting key regulatory pathways of lipid metabolism [34]. Lipoprotein lipase (LPL), a key enzyme in lipid metabolism, hydrolyzes TG contained in circulating chylomicrons and very low‐density lipoproteins (VLDL) into free fatty acids and monoacylglycerols, which are subsequently taken up and utilized by peripheral tissues [35]. Insulin is a major regulator of LPL expression and activity [36]. In states of insulin resistance, LPL expression and activity are suppressed, leading to impaired TG hydrolysis, delayed clearance of chylomicrons and VLDL, and consequent hypertriglyceridemia. Moreover, reduced insulin action accelerates lipolysis in adipose tissue, resulting in increased release of free fatty acids into the circulation [37]. Excess delivery of free fatty acids to the liver promotes hepatic TG synthesis and increases VLDL production and secretion, thereby further aggravating dyslipidemia [8]. Collectively, these metabolic disturbances associated with poor glycemic control contribute to higher TG and LDL‐C levels and lower HDL‐C concentrations [8]. On the other hand, dyslipidemia itself may contribute to the deterioration of glycemic control. Elevated TG levels have been shown to exacerbate insulin resistance, thereby promoting further lipolysis and increasing circulating free fatty acids [13]. This establishes a vicious cycle in which insulin resistance and hypertriglyceridemia reinforce each other, ultimately impairing glucose uptake and worsening glycemic regulation [13]. Additionally, products of TG lipolysis can stimulate hepatic gluconeogenesis, which may further contribute to poor glycemic control [38]. Taken together, these mechanisms provide a reasonable explanation for our findings, whereby increasing values of nontraditional lipid indices derived from TG, LDL‐C, and HDL‐C were associated with higher odds of poor glycemic control.

In the current study, nontraditional lipid indices demonstrated modest but acceptable discriminative ability in identifying poor glycemic control, with AUC values ≥ 0.684. These indices may serve as complementary markers for identifying poor glycemic control in settings where HbA1c measurement is unavailable, costly, or unreliable. Because they are easily calculated from routinely obtained lipid profile parameters, nontraditional lipid indices may have practical utility for risk stratification and preliminary screening of patients with T2D, particularly in resource‐limited settings. Although no statistically significant differences were found among the lipid indices in terms of diagnostic performance, AIP showed the highest AUC (0.687), followed by the TG/HDL‐C ratio (0.686) and LCI (0.686). Previous studies investigating the discriminatory ability of lipid indices for poor glycemic control have reported comparable results, although evidence regarding AIP and LCI is limited. For example, the TG/HDL‐C ratio was shown to have an AUC of 0.620 for predicting poor glycemic control defined as HbA1c ≥ 7% [39]. Another study reported AUCs of 0.760 for CRI‐I, 0.724 for CRI‐II, and 0.695 for the TG/HDL‐C ratio in identifying poor glycemic control (HbA1c > 7%) [31]. Although modest differences in AUC values are present across studies, these variations could be due to differences in the definitions of poor glycemic control, study populations, sample sizes, and statistical methodologies. It is important to emphasize that nontraditional lipid indices are not intended to replace HbA1c assessment; rather, they should be viewed as complementary tools that may help identify patients with T2D who are at higher risk of poor glycemic control, particularly when HbA1c measurement is not feasible. Furthermore, although the levels of nontraditional lipid indices differed significantly between participants with and without poor glycemic control, these differences reflect population‐level associations and may have been influenced by the large sample size. In contrast, the AUC reflects the ability of a marker to distinguish individuals with and without poor glycemic control at the individual level. The modest AUC values observed in this study indicate substantial overlap in the distributions of nontraditional lipid indices between the two groups. Therefore, they should not be viewed as the sole diagnostic tool for poor glycemic control.

This study has several strengths. First, it included a relatively large sample of patients with T2D. Second, it evaluated multiple nontraditional lipid indices, which represent a novel and comprehensive approach, as most previous studies have focused on only one or a limited number of these indices. Third, a comprehensive statistical approach was applied, incorporating RCS, multivariable logistic regression, and ROC analysis to assess all aspects of association and diagnostic performance. Additionally, a wide range of relevant covariates was included in the adjusted models to minimize potential confounding. Nevertheless, it is important to acknowledge several limitations. The cross‐sectional design inhibits the ability to draw conclusions about causality or establish the temporal direction of the observed associations. Residual confounding is also possible, as variables such as physical activity and dietary habits were not investigated and may affect both glycemic control and lipid metabolism. In addition, this was a single‐center study, and the generalizability of the findings to other populations and settings should be interpreted with caution. Therefore, longitudinal and multicenter studies are warranted to clarify the causal relationships between nontraditional lipid indices and poor glycemic control and to evaluate the predictive value of these indices in cohort settings.

## 5. Conclusions

This study showed that several nontraditional lipid indices are significantly associated with poor glycemic control in patients with T2D and demonstrate modest but acceptable discriminatory ability for identifying HbA1c ≥ 7.5%. The observed nonlinear relationships might indicate the close interplay between atherogenic dyslipidemia and glycemic dysregulation. Additionally, given that these indices are derived from routinely measured lipid parameters, they may provide a practical and complementary approach for identifying individuals at higher risk of poor glycemic control, particularly in settings where HbA1c assessment is limited. Future prospective studies are needed to confirm these findings and evaluate causal relationships.

## Author Contributions

Amirhossein Yadegar: methodology, formal analysis, investigation, writing—review and editing, visualization; Fatemeh Mohammadi: methodology, formal analysis, writing—review and editing; Shima Loni: investigation, writing—original draft; Sepideh Yadegar: software, formal analysis, writing—original draft; Ali Mohammadi Naeini: software, investigation, writing—original draft; Amirhossein Tayebi: investigation, writing—original draft; Mahsa Abbaszadeh: validation, writing—review and editing; Soghra Rabizadeh: validation, supervision; Alireza Esteghamati: methodology, data curation, writing—review and editing, supervision; Manouchehr Nakhjavani: methodology, data curation, writing—review and editing, supervision; Sahar Karimpour Reyhan: conceptualization, validation, writing—review and editing, supervision, project administration. Corresponding author had full access to all of the data in this study and takes complete responsibility for the integrity of the data and the accuracy of the data analysis.

## Funding

No funding was received for this manuscript.

## Disclosure

All authors have read and approved the final version of the manuscript.

## Conflicts of Interest

The authors declare no conflicts of interest.

## Supporting Information

Additional supporting information can be found online in the Supporting Information section.

## Supporting information


**Supporting Information 1** Table S1: Lipid indices across HbA1c categories. Table S2: Adjusted odds ratios at selected knots in the restricted cubic spline models. Table S3: Odds ratios of lipid indices for HbA1c ≥ 7.5% in continuous and categorical forms. Table S4: Area under the curve, sensitivity, and specificity from ROC analysis. Table S5: Baseline characteristics of participants according to HbA1c category (<7% vs. ≥7%). Table S6: Associations between lipid indices (continuous and categorical forms) and HbA1c ≥7%. Table S7: Summary of ROC analysis for identifying HbA1c ≥ 7%. Figure S1: RCS analyses show nonlinear associations between lipid indices and HbA1c ≥ 7.5%, indicating increasing trends. The models were adjusted as follows: CRI‐I (diabetes duration, hypertension, WHtR, SBP, DBP, TG, lipid‐lowering agents, and diabetes medications) and CRI‐II (diabetes duration, hypertension, WHtR, SBP, DBP, TG, lipid‐lowering agents, and diabetes medications). Figure S2: Discriminative ability of lipid indices for HbA1c ≥ 7.5% assessed by ROC analysis. The models were adjusted as follows: CRI‐I (diabetes duration, hypertension, WHtR, SBP, DBP, TG, lipid‐lowering agents, and diabetes medications) and CRI‐II (diabetes duration, hypertension, WHtR, SBP, DBP, TG, lipid‐lowering agents, and diabetes medications). Figure S3: Adjusted RCS models with three knots at the 10th, 50th, and 90th percentiles of each index. The models show significant nonlinear associations between all indices and HbA1c ≥ 7%, with higher index values associated with increased odds of HbA1c ≥ 7%. All models were adjusted for diabetes duration, WC, hypertension, SBP, DBP, lipid‐lowering agents, and diabetes medications. Additional covariates were as follows: AC (plus TG), AIP (plus LDL‐C), and TG/HDL‐C ratio (plus LDL‐C). Figure S4: RCS analyses show nonlinear associations between lipid indices and HbA1c ≥ 7%, indicating increasing trends. The models were adjusted as follows: CRI‐I (diabetes duration, hypertension, WC, SBP, DBP, TG, lipid‐lowering agents, and diabetes medications) and CRI‐II (diabetes duration, hypertension, WC, SBP, DBP, TG, lipid‐lowering agents, and diabetes medications). Figure S5: ROC curve analysis showing discriminative ability of lipid indices for HbA1c ≥ 7%. All models were adjusted for diabetes duration, WC, hypertension, SBP, DBP, lipid‐lowering agents, and diabetes medications. Additional covariates were as follows: AC (plus TG), AIP (plus LDL‐C), and TG/HDL‐C ratio (plus LDL‐C). Figure S6: Discriminative ability of lipid indices for HbA1c ≥ 7% assessed by ROC analysis. The models were adjusted as follows: CRI‐I (diabetes duration, hypertension, WC, SBP, DBP, TG, lipid‐lowering agents, and diabetes medications) and CRI‐II (diabetes duration, hypertension, WC, SBP, DBP, TG, lipid‐lowering agents, and diabetes medications).

**Supporting Information 2**  

## Data Availability

The data that support the findings of this study are available from the corresponding author upon reasonable request.
