# Supplementary material for: Association of Nontraditional Lipid Indices With Poor Glycemic Control in Patients With Type 2 Diabetes: A Cross‐Sectional Study
Source: J Diabetes Res. 2026 Sep 8;2026:6993567. doi: 10.1155/jdr/6993567 (PMC13554144; doi:10.1155/jdr/6993567)
Supplement: Supplementary file 1 [file JDR-2026-6993567-s001.pdf]

Association of Non-Traditional Lipid Indices with Poor Glycemic Control in Patients with Type 2 Diabetes: A Cross-Sectional Study

Amirhossein Yadegar, Fatemeh Mohammadi, Shima Loni, Sepideh Yadegar, Ali Mohammadi Naeini, Amirhossein Tayebi, Mahsa Abbaszadeh, Soghra Rabizadeh, Alireza Esteghamati, Manouchehr Nakhjavani, Sahar Karimpour Reyhan

Table S1. Lipid indices across HbA1c categories.

Lipid index	Total (n=6424)	HbA1c<7.5% (n=2967)	HbA1c≥7.5% (n=3457)	P-value
CRI-I	4.15±1.27	4.04±1.23	4.24±1.29	<0.001
CRI-II	2.35±0.92	2.29±0.90	2.40±0.94	<0.001

Data are presented as mean ± SD.

HbA1c: hemoglobin A1C; CRI-I: Castelli risk index-I; CRI-II: Castelli risk index-II.

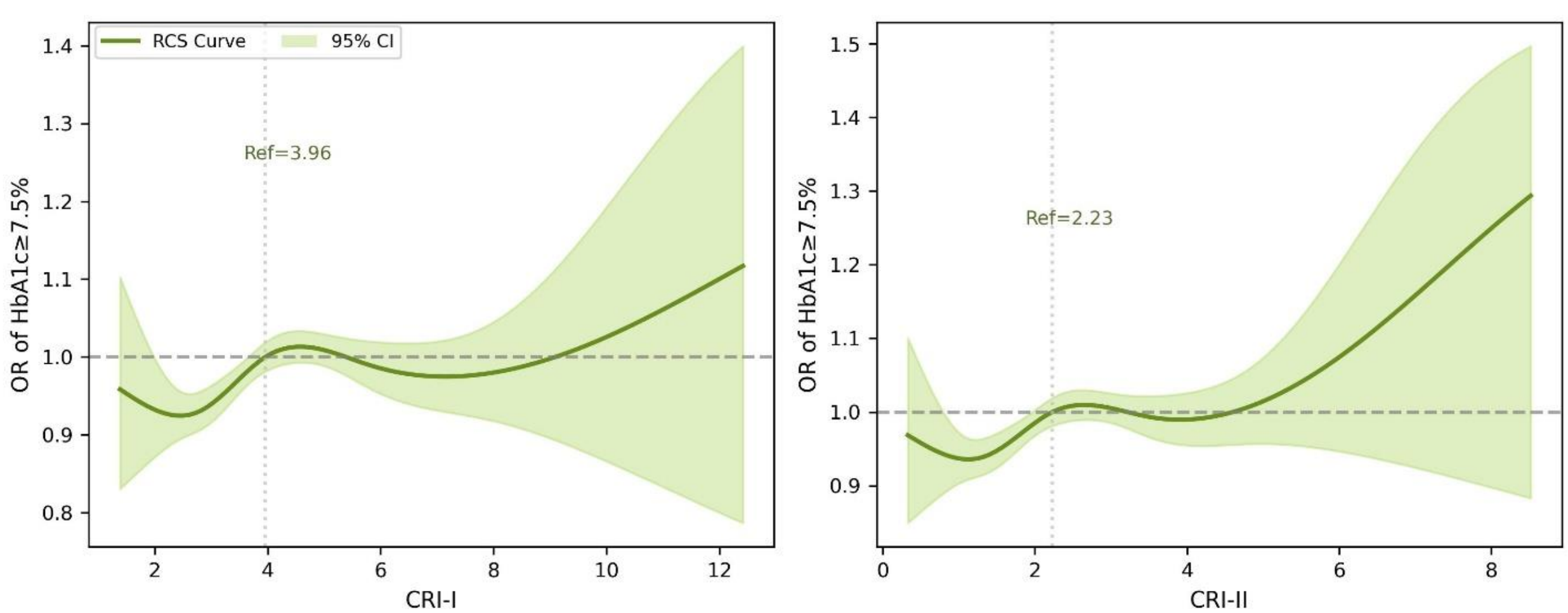

Figure S1. RCS analyses show non-linear associations between lipid indices and HbA1c≥7.5%, indicating increasing trends. The models were adjusted as follows: CRI-I (diabetes duration, hypertension, WHtR, SBP, DBP, TG, lipid-lowering agents, and diabetes medications) and CRI-II (diabetes duration, hypertension, WHtR, SBP, DBP, TG, lipid-lowering agents, and diabetes medications).

RCS: restricted cubic spline; CI: confidence interval; OR: odds ratio; HbA1c: hemoglobin A1C; CRI-I: Castelli risk index-I; CRI-II: Castelli risk index-II; WHtR: waist-to-height ratio; SBP: systolic blood pressure; DBP: diastolic blood pressure; TG: triglycerides.

Table S2. Adjusted odds ratios at selected knots in the restricted cubic spline models.

Lipid index	Selected knots			
	10th percentile		90th percentile	
	Value	Adjusted OR (95% CI) [a]	Value	Adjusted OR (95% CI) [a]
CRI-I	2.75	0.93 (0.90-0.95)	5.81	0.99 (0.96-1.02)
CRI-II	1.30	0.94 (0.91-0.96)	3.53	0.99 (0.96-1.02)

[a]: The models were adjusted as follows: CRI-I (diabetes duration, hypertension, WHtR, SBP, DBP, TG, lipid-lowering agents, and diabetes medications) and CRI-II (diabetes duration, hypertension, WHtR, SBP, DBP, TG, lipid-lowering agents, and diabetes medications).

CRI-I: Castelli risk index-I; CRI-II: Castelli risk index-II; OR: odds ratio; CI: confidence interval; WHtR: waist-to-height ratio; SBP: systolic blood pressure; DBP: diastolic blood pressure; TG: triglycerides.

Table S3. Odds ratios of lipid indices for HbA1c≥7.5% in continuous and categorical forms.

Lipid index		OR (95% CI) of HbA1c≥7.5%	
		Unadjusted	Adjusted [a]
CRI-I	Continuous (Per one unit)	1.13 (1.09-1.18)	1.07 (1.02-1.12)
	Q1	Ref	
	Q2	1.26 (1.09–1.44)	1.25 (1.08-1.45)
	Q3	1.50 (1.30–1.72)	1.43 (1.24-1.66)
	Q4	1.51 (1.31–1.73)	1.26 (1.07-1.48)
CRI-II	Continuous (Per one unit)	1.13 (1.07-1.19)	1.10 (1.03-1.16)
	Q1	Ref	
	Q2	1.15 (1.00–1.32)	1.16 (1.00-1.33)
	Q3	1.33 (1.16–1.53)	1.34 (1.16-1.55)
	Q4	1.35 (1.18–1.55)	1.26 (1.08-1.46)

[a]: The models were adjusted as follows: CRI-I (diabetes duration, hypertension, WHtR, SBP, DBP, TG, lipid-lowering agents, and diabetes medications) and CRI-II (diabetes duration, hypertension, WHtR, SBP, DBP, TG, lipid-lowering agents, and diabetes medications).

HbA1c: hemoglobin A1C; OR: odds ratio; CI: confidence interval; CRI-I: Castelli risk index-I; CRI-II: Castelli risk index-II; WHtR: waist-to-height ratio; SBP: systolic blood pressure; DBP: diastolic blood pressure; TG: triglycerides.

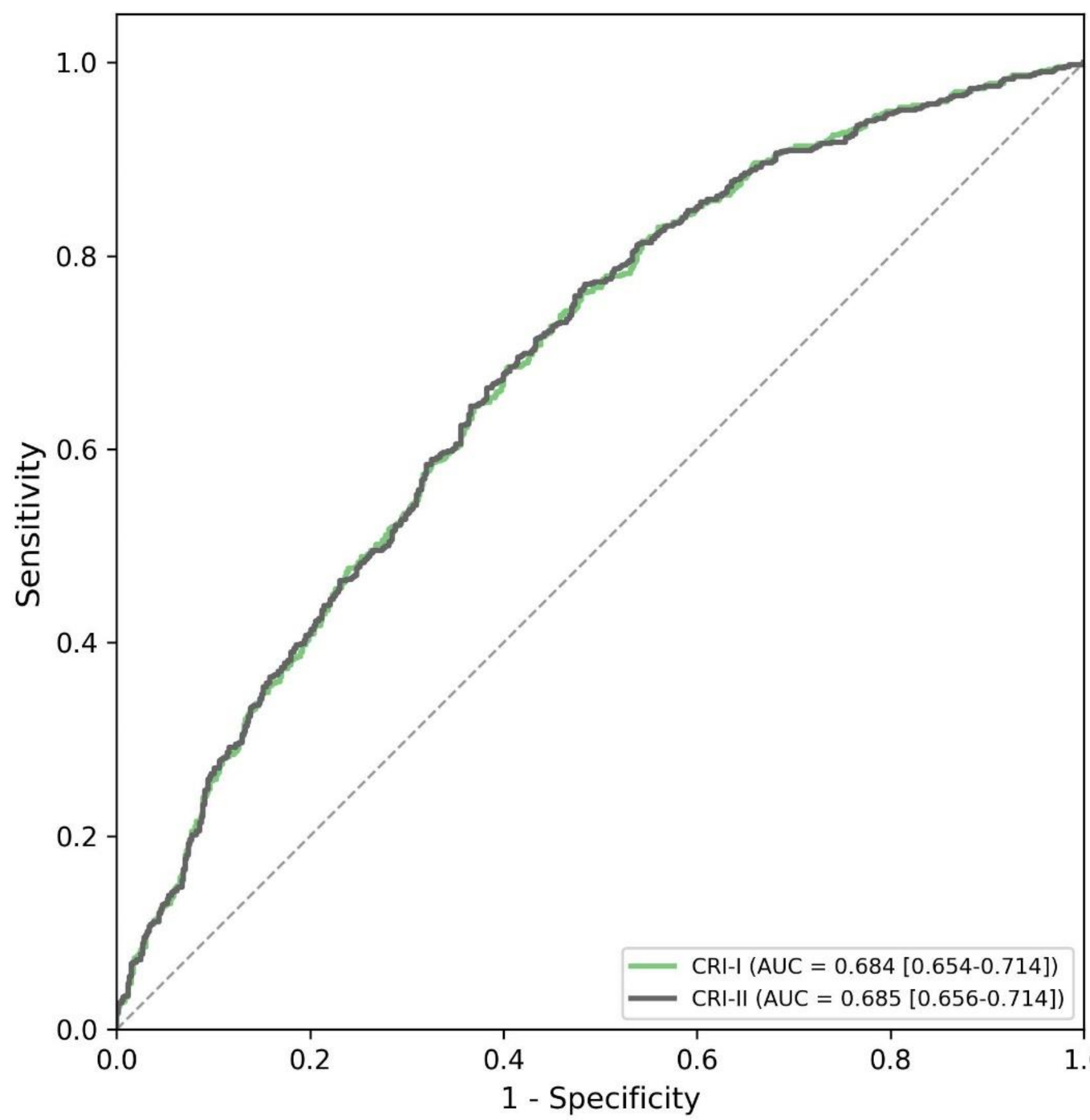

Figure S2. Discriminative ability of lipid indices for HbA1c≥7.5% assessed by ROC analysis. The models were adjusted as follows: CRI-I (diabetes duration, hypertension, WHtR, SBP, DBP, TG, lipid-lowering agents, and diabetes medications) and CRI-II (diabetes duration, hypertension, WHtR, SBP, DBP, TG, lipid-lowering agents, and diabetes medications).

HbA1c: hemoglobin A1C; ROC: receiver operating characteristic; AUC: area under the curve; CRI-I: Castelli risk index-I; CRI-II: Castelli risk index-II; WHtR: waist-to-height ratio; SBP: systolic blood pressure; DBP: diastolic blood pressure; TG: triglycerides.

Table S4. Area under the curve, sensitivity, and specificity from ROC analysis.

Lipid index	Cutoff	AUC (95%CI) [a]	Sensitivity	Specificity	Accuracy	P-value
CRI-I	3.98	0.684 (0.654-0.714)	76%	52%	65%	<0.001
CRI-II	2.25	0.685 (0.656-0.714)	77%	52%	65%	<0.001

[a]: The models were adjusted as follows: CRI-I (diabetes duration, hypertension, WHtR, SBP, DBP, TG, lipid-lowering agents, and diabetes medications) and CRI-II (diabetes duration, hypertension, WHtR, SBP, DBP, TG, lipid-lowering agents, and diabetes medications).

ROC: receiver operating characteristic; AUC: area under the curve; CI: confidence interval; CRI-I: Castelli risk index-I; CRI-II: Castelli risk index-II; WHtR: waist-to-height ratio; SBP: systolic blood pressure; DBP: diastolic blood pressure; TG: triglycerides.

Table S5. Baseline characteristics of participants according to HbA1c category (<7% vs. ≥7%).

Variable		HbA1c<7% (n=2125)	HbA1c≥7% (n=4299)	P-value
Age (years)		58.0±10.8	57.6±10.5	0.131
Diabetes duration (years)		6 (3-10)	9 (4-15)	<0.001
Women (n, %)		1236 (58.2%)	2397 (55.8%)	0.067
Hypertension (n, %)		870 (40.9%)	1897 (44.1%)	0.015
BMI (Kg/m^2)		28.8±4.7	28.9±4.7	0.273
WC (cm)		97.4±9.9	98.0±10.3	0.036
WHtR		0.60±0.07	0.61±0.07	0.053
SBP (mmHg)		128.8±16.7	131.4±18.3	<0.001
DBP (mmHg)		78.3±9.1	79.2±9.5	<0.001
FPG (mg/dL)		130.1±32.0	183.1±64.4	<0.001
2hPG (mg/dL)		174.8±59.1	256.3±92.1	<0.001
TG (mg/dL)		138 (102-191)	155 (111-220)	<0.001
TC (mg/dL)		173.3±40.8	181.2±46.2	<0.001
LDL-C (mg/dL)		97.7±32.6	102.1±35.7	<0.001
HDL-C (mg/dL)		45.1±11.2	44.8±11.1	0.239
Non-HDL-C (mg/dL)		128.2±39.7	136.4±44.4	<0.001
eGFR (mL/min/1.73m²)		77.7±17.3	77.9±17.6	0.699
Lipid-lowering agents (n, %)	Atorvastatin	1827 (86.0%)	3602 (83.8%)	0.066
	Rosuvastatin	237 (11.2%)	564 (13.1%)	
	Fibrates	61 (2.9%)	133 (3.1%)	
Diabetes medications (n, %)	Metformin monotherapy	1521 (71.6%)	2272 (52.8%)	<0.001
	Sulfonylurea monotherapy	89 (4.2%)	251 (5.8%)	
	Insulin monotherapy	6 (0.3%)	27 (0.6%)	
	Multiple drug therapy	509 (24.0%)	1749 (40.7%)	
AC		3.02±1.22	3.22±1.29	<0.001
AIP		0.14 (-0.02-0.32)	0.19 (0.03-0.37)	<0.001
LCI		13005 (8642-20113)	15111 (9408-23736)	<0.001
TG/HDL-C ratio		3.14 (2.17-4.80)	3.54 (2.44-5.35)	<0.001
CRI-I		4.02±1.22	4.22±1.29	<0.001
CRI-II		2.28±0.91	2.38±0.93	<0.001

Continuous variables are presented as mean ± SD or median (Q1–Q3); categorical variables as n (%).

HbA1c: hemoglobin A1C; BMI: body mass index; WC: waist circumference; WHtR: waist-to-height ratio; SBP: systolic blood pressure; DBP: diastolic blood pressure; FPG: fasting plasma glucose; 2hPG: 2-hour postprandial glucose; TG: triglycerides; TC: total cholesterol; LDL-C: low-density lipoprotein cholesterol; HDL-C: high-density lipoprotein cholesterol; non-HDL-C: non-high-density lipoprotein cholesterol; eGFR:

estimated glomerular filtration rate; AC: atherogenic coefficient; AIP: atherogenic index of plasma; LCI: lipid combine index; Castelli risk index-I; CRI-II: Castelli risk index-II.

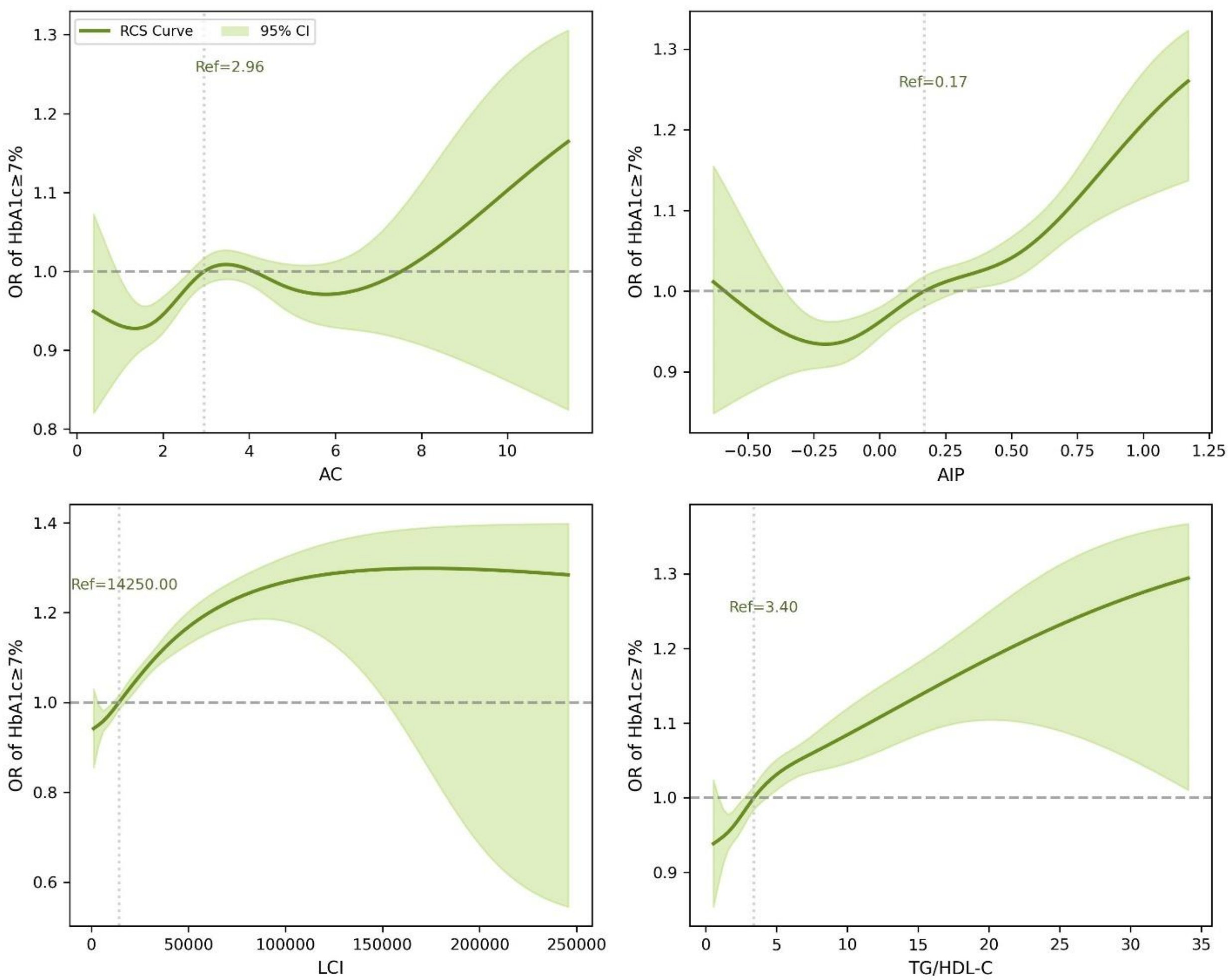

Figure S3. Adjusted RCS models with three knots at the 10th, 50th, and 90th percentiles of each index. The models show significant non-linear associations between all indices and HbA1c≥7%, with higher index values associated with increased odds of HbA1c≥7%. All models were adjusted for diabetes duration, WC, hypertension, SBP, DBP, lipid-lowering agents, and diabetes medications. Additional covariates were as follows: AC (plus TG), AIP (plus LDL-C), and TG/HDL-C ratio (plus LDL-C).

RCS: restricted cubic spline; CI: confidence interval; OR: odds ratio; HbA1c: hemoglobin A1C; AC: atherogenic coefficient; AIP: atherogenic index of plasma; LCI: lipid combine index; TG: triglycerides; HDL-C: high-density lipoprotein cholesterol; SBP: systolic blood pressure; DBP: diastolic blood pressure; WC: waist circumference; LDL-C: low-density lipoprotein cholesterol.

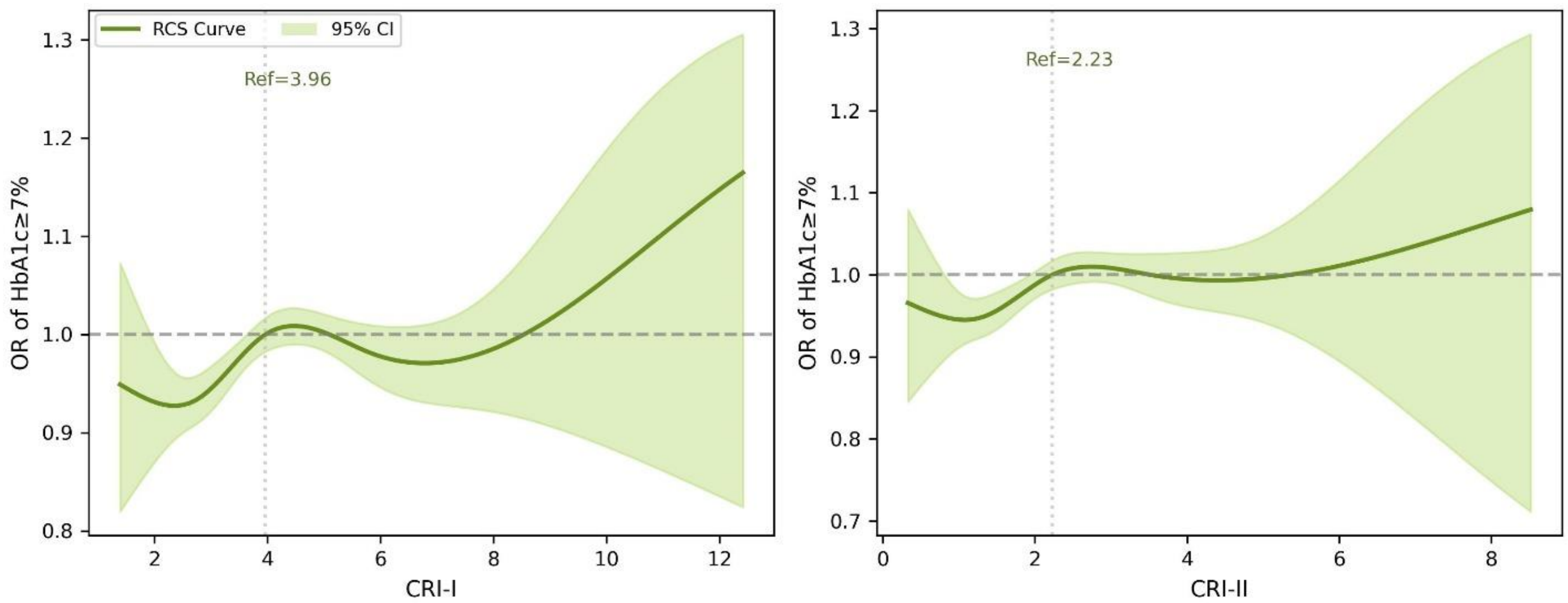

Figure S4. RCS analyses show non-linear associations between lipid indices and HbA1c≥7%, indicating increasing trends. The models were adjusted as follows: CRI-I (diabetes duration, hypertension, WC, SBP, DBP, TG, lipid-lowering agents, and diabetes medications) and CRI-II (diabetes duration, hypertension, WC, SBP, DBP, TG, lipid-lowering agents, and diabetes medications).

RCS: restricted cubic spline; CI: confidence interval; OR: odds ratio; HbA1c: hemoglobin A1C; CRI-I: Castelli risk index-I; CRI-II: Castelli risk index-II; WC: waist circumference; SBP: systolic blood pressure; DBP: diastolic blood pressure; TG: triglycerides.

Table S6. Associations between lipid indices (continuous and categorical forms) and HbA1c≥7%.

Lipid index		OR (95% CI) of HbA1c≥7%	
		Unadjusted	Adjusted [a]
AC	Continuous (Per one unit)	1.14 (1.09-1.19)	1.07 (1.01-1.13)
	Q1	Ref	
	Q2	1.20 (1.04–1.38)	1.18 (1.02-1.37)
	Q3	1.51 (1.30–1.74)	1.43 (1.22-1.67)
	Q4	1.46 (1.26–1.69)	1.20 (1.01-1.43)
AIP	Continuous (Per one unit)	2.19 (1.78-2.68)	2.18 (1.75-2.70)
	Q1	Ref	
	Q2	1.23 (1.06–1.41)	1.23 (1.06-1.43)
	Q3	1.44 (1.24–1.66)	1.41 (1.21-1.65)
	Q4	1.70 (1.47–1.98)	1.70 (1.45-1.99)
LCI	Continuous (Per 1000 unit)	1.02 (1.02-1.02)	1.02 (1.02-1.03)
	Q1	Ref	
	Q2	1.02 (0.89–1.18)	1.10 (0.94-1.27)
	Q3	1.22 (1.06–1.41)	1.32 (1.13-1.53)
	Q4	1.69 (1.45–1.96)	1.86 (1.59-2.18)
TG/HDL-C ratio	Continuous (Per one unit)	1.07 (1.05-1.09)	1.07 (1.05-1.10)
	Q1	Ref	
	Q2	1.25 (1.08–1.44)	1.26 (1.08-1.46)
	Q3	1.46 (1.26–1.69)	1.45 (1.24-1.69)
	Q4	1.72 (1.48–1.99)	1.72 (1.47-2.01)
CRI-I	Continuous (Per one unit)	1.14 (1.09-1.19)	1.07 (1.01-1.13)
	Q1	Ref	
	Q2	1.20 (1.04–1.38)	1.18 (1.02-1.37)
	Q3	1.51 (1.30–1.74)	1.43 (1.22-1.67)
	Q4	1.46 (1.26–1.69)	1.20 (1.01-1.43)
CRI-II	Continuous (Per one unit)	1.13 (1.06-1.19)	1.09 (1.02-1.16)
	Q1	Ref	
	Q2	1.07 (0.93–1.24)	1.07 (0.92-1.24)
	Q3	1.28 (1.10–1.48)	1.27 (1.09-1.49)
	Q4	1.30 (1.13–1.51)	1.21 (1.03-1.42)

[a]: All models were adjusted for diabetes duration, WC, hypertension, SBP, DBP, lipid-lowering agents, and diabetes medications. Additional covariates were as follows: AC (plus TG), AIP (plus LDL-C), TG/HDL-C ratio (plus LDL-C), CRI-I (plus TG), and CRI-II (plus TG).

HbA1c: hemoglobin A1C; OR: odds ratio; CI: confidence interval; AC: atherogenic coefficient; AIP: atherogenic index of plasma; LCI: lipid combine index; TG: triglycerides; HDL-C: high-density lipoprotein cholesterol; CRI-I: Castelli risk index-I; CRI-II: Castelli risk index-II; SBP: systolic blood pressure; DBP: diastolic blood pressure; WC: waist circumference; LDL-C: low-density lipoprotein cholesterol.

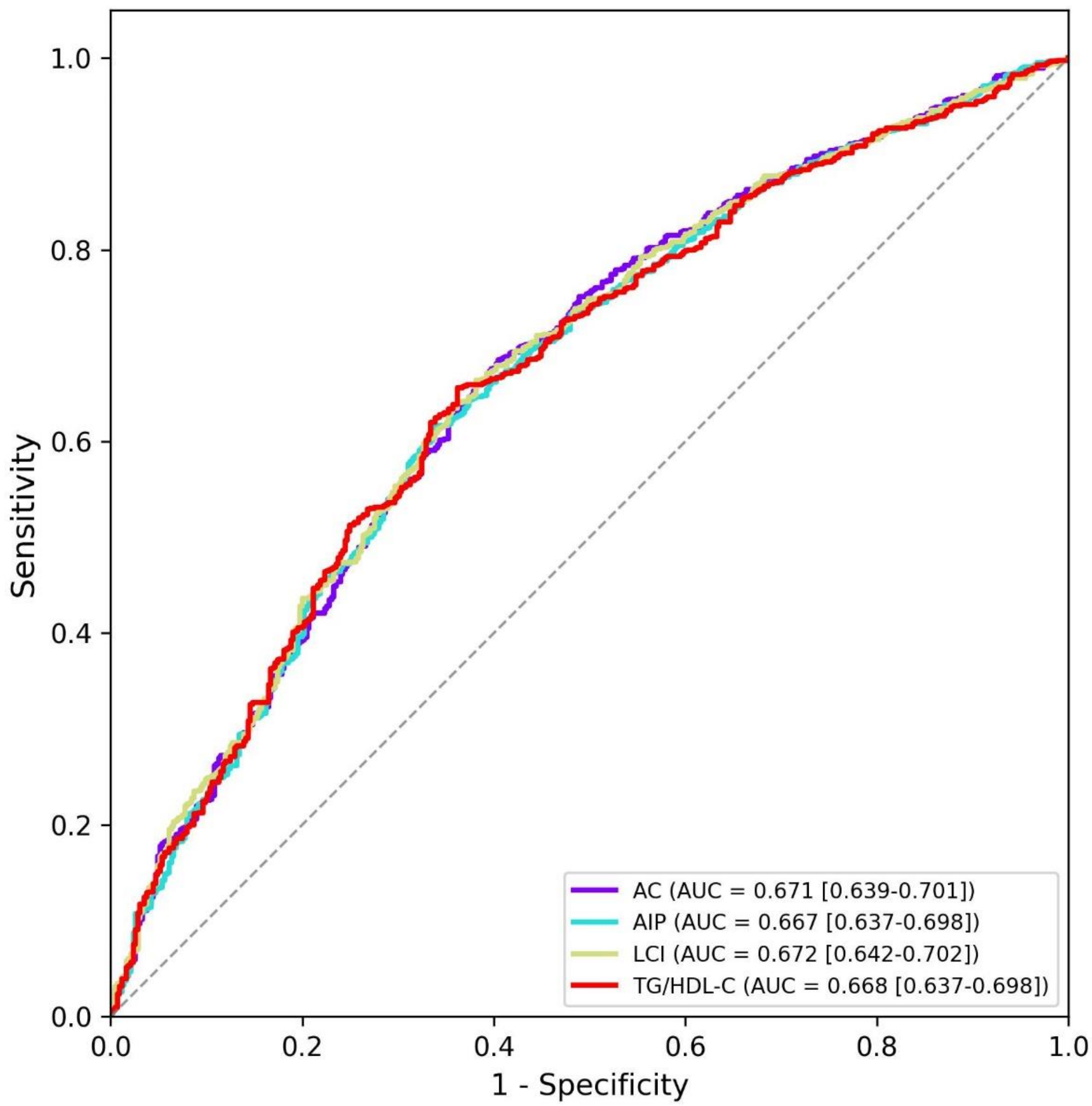

Figure S5. ROC curve analysis showing discriminative ability of lipid indices for HbA1c≥7%. All models were adjusted for diabetes duration, WC, hypertension, SBP, DBP, lipid-lowering agents, and diabetes medications. Additional covariates were as follows: AC (plus TG), AIP (plus LDL-C), and TG/HDL-C ratio (plus LDL-C).

ROC: receiver operating characteristic; AUC: area under the curve; HbA1c: hemoglobin A1C; AC: atherogenic coefficient; AIP: atherogenic index of plasma; LCI: lipid combine index; TG: triglycerides; HDL-C: high-density lipoprotein cholesterol; SBP: systolic blood pressure; DBP: diastolic blood pressure; WC: waist circumference; LDL-C: low-density lipoprotein cholesterol.

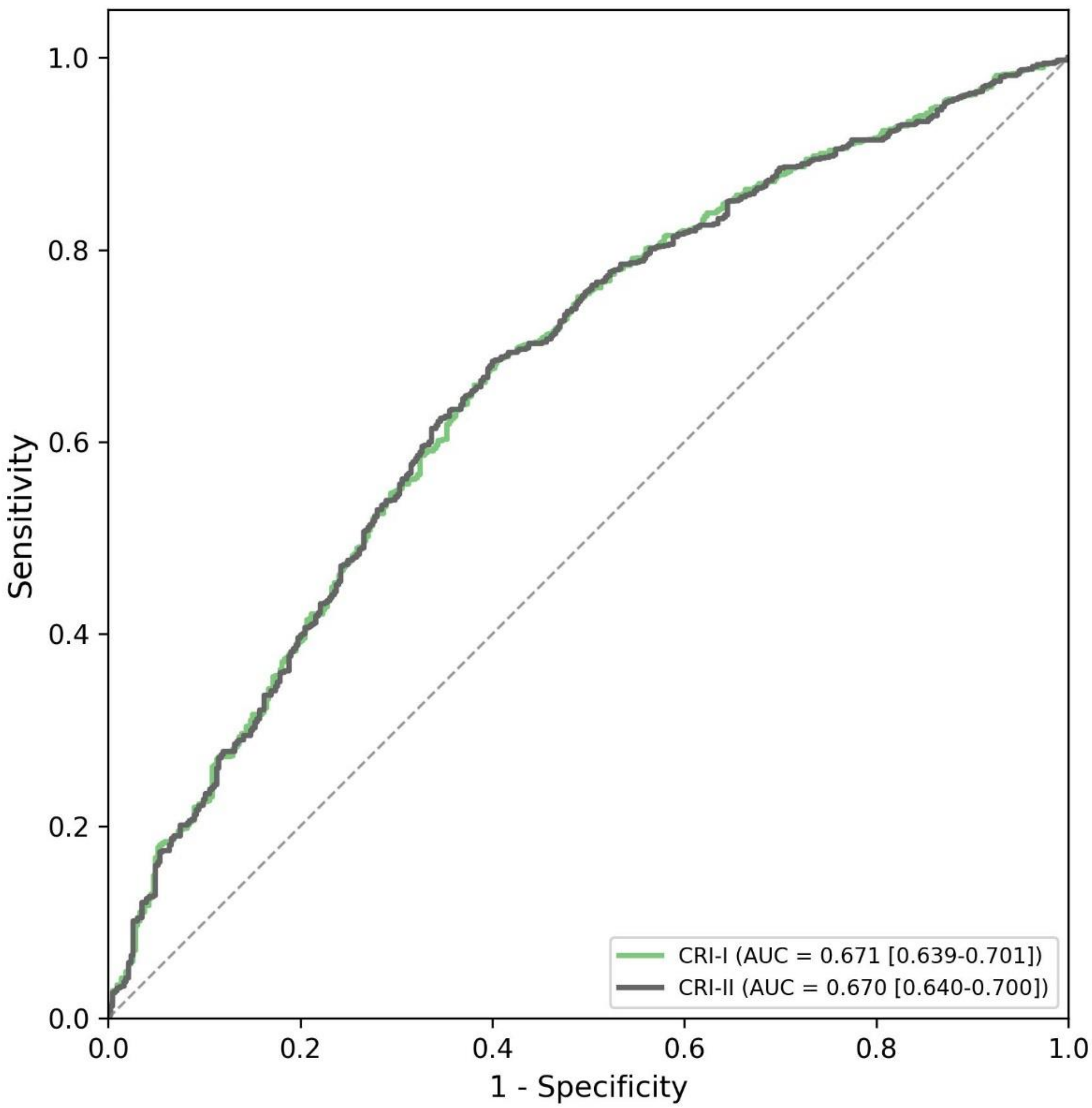

Figure S6. Discriminative ability of lipid indices for HbA1c≥7% assessed by ROC analysis. The models were adjusted as follows: CRI-I (diabetes duration, hypertension, WC, SBP, DBP, TG, lipid-lowering agents, and diabetes medications) and CRI-II (diabetes duration, hypertension, WC, SBP, DBP, TG, lipid-lowering agents, and diabetes medications).

HbA1c: hemoglobin A1C; ROC: receiver operating characteristic; AUC: area under the curve; CRI-I: Castelli risk index-I; CRI-II: Castelli risk index-II; WC: waist circumference; SBP: systolic blood pressure; DBP: diastolic blood pressure; TG: triglycerides.

Table S7. Summary of ROC analysis for identifying HbA1c≥7%.

Lipid indices	Cutoff	AUC (95%CI) [a]	Sensitivity	Specificity	Accuracy	P-value
AC	3.25	0.671 (0.639-0.701)	68%	60%	66%	<0.001
AIP	0.20	0.667 (0.637-0.698)	62%	66%	63%	<0.001
LCI	13720	0.672 (0.642-0.702)	66%	62%	65%	<0.001
TG/HDL-C ratio	3.77	0.668 (0.637-0.698)	66%	64%	65%	<0.001
CRI-I	4.25	0.671 (0.639-0.701)	68%	60%	66%	<0.001
CRI-II	2.35	0.670 (0.640-0.700)	68%	60%	66%	<0.001

[a]: All models were adjusted for diabetes duration, WC, hypertension, SBP, DBP, lipid-lowering agents, and diabetes medications. Additional covariates were as follows: AC (plus TG), AIP (plus LDL-C), TG/HDL-C ratio (plus LDL-C), CRI-I (plus TG), and CRI-II (plus TG).

ROC: receiver operating characteristic; HbA1c: hemoglobin A1C; AUC: area under the curve; CI: confidence interval; AC: atherogenic coefficient; AIP: atherogenic index of plasma; LCI: lipid combine index; TG: triglycerides; HDL-C: high-density lipoprotein cholesterol; CRI-I: Castelli risk index-I; CRI-II: Castelli risk index-II; SBP: systolic blood pressure; DBP: diastolic blood pressure; WC: waist circumference; LDL-C: low-density lipoprotein cholesterol.
