# Supplementary material for: Association of Nontraditional Lipid Indices With Poor Glycemic Control in Patients With Type 2 Diabetes: A Cross‐Sectional Study
Source: J Diabetes Res. 2026 Sep 8;2026:6993567. doi: 10.1155/jdr/6993567 (PMC13554144; doi:10.1155/jdr/6993567)
Supplement: Supplementary file 2 [file JDR-2026-6993567-s002.docx]

STROBE Statement—checklist of items that should be included in reports of observational studies

|  | Item No. | Recommendation | Page  No. | Relevant text from manuscript |
| --- | --- | --- | --- | --- |
| **Title and abstract** | 1 | (*a*) Indicate the study’s design with a commonly used term in the title or the abstract | 1 | Association of Non-Traditional Lipid Indices with Poor Glycemic Control in Patients with Type 2 Diabetes: A Cross-Sectional Study |
|  |  | (*b*) Provide in the abstract an informative and balanced summary of what was done and what was found | 2-3 | Background: This study investigated the association between poor glycemic control… |
| Introduction | | | |  |
| Background/rationale | 2 | Explain the scientific background and rationale for the investigation being reported | 3-4 | As per projections from the International Diabetes Federation (IDF)… |
| Objectives | 3 | State specific objectives, including any prespecified hypotheses | 4-5 | Therefore, this study sought to investigate… |
| Methods | | | |  |
| Study design | 4 | Present key elements of study design early in the paper | 5 | This cross-sectional research… |
| Setting | 5 | Describe the setting, locations, and relevant dates, including periods of recruitment, exposure, follow-up, and data collection | 5 | This cross-sectional research included individuals with T2D who visited the diabetes clinic of a tertiary hospital affiliated with Tehran University of Medical Sciences from 2014 to 2024. |
| Participants | 6 | (*a*) *Cohort study*—Give the eligibility criteria, and the sources and methods of selection of participants. Describe methods of follow-up  *Case-control study*—Give the eligibility criteria, and the sources and methods of case ascertainment and control selection. Give the rationale for the choice of cases and controls  *Cross-sectional study*—Give the eligibility criteria, and the sources and methods of selection of participants | 5 | Exclusion criteria included conditions that… |
|  |  | (*b*) *Cohort study*—For matched studies, give matching criteria and number of exposed and unexposed  *Case-control study*—For matched studies, give matching criteria and the number of controls per case |  |  |
| Variables | 7 | Clearly define all outcomes, exposures, predictors, potential confounders, and effect modifiers. Give diagnostic criteria, if applicable | 6 | Poor glycemic control was defined as HbA1c≥7.5%... |
| Data sources/ measurement | 8* | For each variable of interest, give sources of data and details of methods of assessment (measurement). Describe comparability of assessment methods if there is more than one group | 5-6 | Demographic and clinical details, such as age, gender, diabetes duration, medical history, and medication use, were gathered from medical records… |
| Bias | 9 | Describe any efforts to address potential sources of bias | 5 | The study population was relatively homogeneous… |
| Study size | 10 | Explain how the study size was arrived at | 5 | This cross-sectional research included individuals with T2D who visited… |

Continued on next page

| Quantitative variables | 11 | Explain how quantitative variables were handled in the analyses. If applicable, describe which groupings were chosen and why | 7 | Variables that follow a normal distribution were expressed as mean ± standard deviation (SD), while those that do not conform to a normal distribution were reported using median (Q1, Q3). |
| --- | --- | --- | --- | --- |
| Statistical methods | 12 | (*a*) Describe all statistical methods, including those used to control for confounding | 7-8 | To investigate the association between non-traditional lipid indices and poor glycemic control (HbA1c≥7.5%), restricted cubic spline (RCS) models… |
|  |  | (*b*) Describe any methods used to examine subgroups and interactions | 7 | Multivariable logistic regression analyses were conducted to examine the association between HbA1c≥7.5% and both continuous and categorical (quartile-based) forms of each lipid index… |
|  |  | (*c*) Explain how missing data were addressed | 7 | The Kolmogorov–Smirnov test, along with visual inspections… |
|  |  | (*d*) *Cohort study*—If applicable, explain how loss to follow-up was addressed  *Case-control study*—If applicable, explain how matching of cases and controls was addressed  *Cross-sectional study*—If applicable, describe analytical methods taking account of sampling strategy | NA |  |
|  |  | (*e*) Describe any sensitivity analyses | 8 | As a sensitivity analysis, all primary analyses were repeated… |
| Results | | | | |
| Participants | 13* | (a) Report numbers of individuals at each stage of study—eg numbers potentially eligible, examined for eligibility, confirmed eligible, included in the study, completing follow-up, and analysed | 8 | This study enrolled 6424 individuals with T2D… |
|  |  | (b) Give reasons for non-participation at each stage | 8 | This study enrolled 6424 individuals with T2D… |
|  |  | (c) Consider use of a flow diagram | NA |  |
| Descriptive data | 14* | (a) Give characteristics of study participants (eg demographic, clinical, social) and information on exposures and potential confounders | 8 | with a mean age of 57.7±10.6 years. The median diabetes duration was 8 years (Q1-Q3: 4-14), and 56.6% were women (n=3633)… |
|  |  | (b) Indicate number of participants with missing data for each variable of interest | NA |  |
|  |  | (c) *Cohort study*—Summarise follow-up time (eg, average and total amount) | NA |  |
| Outcome data | 15* | *Cohort study*—Report numbers of outcome events or summary measures over time | NA |  |
|  |  | *Case-control study—*Report numbers in each exposure category, or summary measures of exposure | NA |  |
|  |  | *Cross-sectional study—*Report numbers of outcome events or summary measures | 8 | Compared with patients with HbA1c<7.5%, those with HbA1c≥7.5%... |
| Main results | 16 | (*a*) Give unadjusted estimates and, if applicable, confounder-adjusted estimates and their precision (eg, 95% confidence interval). Make clear which confounders were adjusted for and why they were included | 12 | Multivariable logistic regression analyses were conducted… |
|  |  | (*b*) Report category boundaries when continuous variables were categorized | Table 2 |  |
|  |  | (*c*) If relevant, consider translating estimates of relative risk into absolute risk for a meaningful time period | NA |  |

Continued on next page

| Other analyses | 17 | Report other analyses done—eg analyses of subgroups and interactions, and sensitivity analyses | 15 | Sensitivity analyses using HbA1c ≥7.0% showed findings… |
| --- | --- | --- | --- | --- |
| Discussion | | | | |
| Key results | 18 | Summarise key results with reference to study objectives | 15 | This study evaluated the association between poor glycemic control… |
| Limitations | 19 | Discuss limitations of the study, taking into account sources of potential bias or imprecision. Discuss both direction and magnitude of any potential bias | 19-20 | Nevertheless, it is important to acknowledge several limitations. The cross-sectional design inhibits… |
| Interpretation | 20 | Give a cautious overall interpretation of results considering objectives, limitations, multiplicity of analyses, results from similar studies, and other relevant evidence | 20 | This study showed that several non-traditional lipid indices are significantly associated… |
| Generalisability | 21 | Discuss the generalisability (external validity) of the study results | 20 | they may provide a practical and complementary approach for identifying individuals at higher risk of poor glycemic control, particularly in settings where HbA1c… |
| Other information | |  | | |
| Funding | 22 | Give the source of funding and the role of the funders for the present study and, if applicable, for the original study on which the present article is based | 21 | This study did not receive any specific funding. |

*Give information separately for cases and controls in case-control studies and, if applicable, for exposed and unexposed groups in cohort and cross-sectional studies.

**Note:** An Explanation and Elaboration article discusses each checklist item and gives methodological background and published examples of transparent reporting. The STROBE checklist is best used in conjunction with this article (freely available on the Web sites of PLoS Medicine at http://www.plosmedicine.org/, Annals of Internal Medicine at http://www.annals.org/, and Epidemiology at http://www.epidem.com/). Information on the STROBE Initiative is available at www.strobe-statement.org.
